# TRPM4 Inhibition: An Unexpected Mechanism of NO-Induced Vasodilatation

**DOI:** 10.1093/function/zqac007

**Published:** 2022-03-24

**Authors:** Michael J Davis

**Affiliations:** Department of Medical Pharmacology and Physiology, University of Missouri School of Medicine, Columbia 65212, MO, USA

## A Perspective on “Nitric Oxide Signals Through IRAG to Inhibit TRPM4 Channels and Dilate Cerebral Arteries”

Nitric oxide (NO) is produced from the endothelium in response to shear stress and/or agonist stimulation. Nitric oxide diffuses to the overlying smooth muscle layer of blood vessels, where it stimulates soluble guanylyl cyclase (sGC) to catalyze the intracellular conversion of cGMP from GTP; cGMP then stimulates the activity of cGMP-dependent protein kinases (PKGs), which in turn phosphorylate target proteins in smooth muscle to induce relaxation. The pioneering work by Furchgott, Ignarro, and Murad to elucidate how the NO/cGMP/PKG signaling pathway produces vasodilatation was awarded the 1998 Nobel Prize in Physiology/Medicine.

Arterial dilatation via NO/cGMP/PKG signaling is associated with a reduction in cytoplasmic calcium level, leading to dephosphorylation of the regulatory myosin light chain (MLC_20_) and inhibition of actomyosin shortening. Multiple targets of PKG family kinases in vascular smooth muscle (VSM), include ion channels, calcium regulatory proteins, and contractile filaments. Potential ion channel targets include calcium-activated K^+^ (KCa) channels and voltage-gated calcium channels (VGCCs). Nitric oxide-mediated activation of a K^+^ channel would lead to VSM hyperpolarization,^1^ reducing calcium entry through VGCCs to inhibit contraction; alternatively, NO/cGMP/PKG signaling could directly inhibit VGCC activity by phosphorylation of specific cytoplasmic channel residues that influence gating.^2^^,^^3^ However, studies on ion channel targets of NO signaling in VSM have resulted in conflicting conclusions by various laboratories.^4^^,^^5^

In this issue of *FUNCTION*, Ali et al. present evidence that TRPM4 channels are a primary target of the NO/cGMP/PKG signaling pathway in mouse cerebral vascular smooth muscle.^6^ TRPM4 is a member of the melastatin family of TRP (transient receptor potential channels) that is permeable to monovalent cations and activated by intracellular calcium. At the normal VSM resting potential, TRPM4 activation leads to net Na^+^ influx and depolarization that enhances voltage-dependent influx of calcium through L-type VGCCs to promote MLC_20_ phosphorylation. Previous work by this laboratory (Earley and colleagues) established that TRPM4 activity mediates a substantial degree of pressure-dependent myogenic tone in rat and mouse cerebral arteries, maintaining arterial diameter (and thus blood flow) at an intermediate level so that it can be modulated in either direction by endothelial and neural influences (for review see^7^). Patch-clamp studies of freshly isolated cerebral artery myocytes in this and previous studies show that TRPM4 channels can be activated by pipette suction in the perforated patch recording mode (which allows intracellular calcium levels to change), producing “transient inward cation currents” (TICCs).^6^^,^^7^ Unfortunately, only limited tools are available for definitively determining that these currents are conducted only by TRPM4 channels, such as their inhibition by 9-phenanthrol, which has off-target effects on other cation channels as well as the calcium-activated Cl^−^ channel Anoctoamin1 (TMEM16A).^8^ However, Earley and colleagues have previously shown that the concentration of 9-phenanthrol used to block TRPM4 current in cerebral artery myocytes does not substantially alter currents mediated by TRPC3/C6, voltage-gated K^+^, BKCa, K_IR_, or L-type VGCC channels,^7^ and ion substitution protocols in the present study confirm that calcium-activated TICCs do indeed represent cation influx rather than Cl^−^ efflux.^6^

The pressure-dependency of TRPM4 channel activity results from the activation of phospholipase C (PLC) downstream from G-proteins that may be both mechano- and agonist sensitive. In cerebral VSM, PLCγ1 catalyzes the production of diacylglycerol (DAG) and IP_3_ from phosphatidylinositol 4,5-bisphosphate in the plasma membrane.^7^ In this new study, Ali et al.^6^ show that stretch-induced TICC activity in isolated cerebral artery myocytes is inhibited by application of the NO donor S-nitroso-N-acetylpencillamine (SNAP) or by the membrane-permeable cGMP analog, dibutyryl-cGMP. Conversely, SNAP has no effect on stretch-induced TICC activity in the presence of sGC or PKG blockade.

How might NO inhibit TRPM4 activity? The possibility that NO directly inhibits the TRPM4 channel was ruled down by the finding that whole-cell TRPM4 currents were unaffected by SNAP under conditions in which intracellular calcium levels were clamped. Because the primary means of TRPM4 activation in VSM is mediated by IP_3_-mediated Ca^2+^ release from sarcoplasmic reticulum (SR),^7^ Ali et al. tested the possible role of IP_3_R-associated cGMP kinase substrate (IRAG) as a phosphorylation target of PKG. IRAG protein expression in mouse cerebral arteries was confirmed by capillary electrophoresis. Knock down of IRAG by ∼40% using morpholino oligonucleotides in mouse cerebral arteries maintained in short-term organ-culture substantially impaired their subsequent dilation to an NO donor without directly affecting myogenic tone.^6^

A final aspect of this study was to test the association of IRAG and PKG with IP_3_ receptors (IP_3_Rs). As established for other ion channel regulatory complexes, these proteins are presumably clustered in a preformed signaling complex to facilitate their interactions. Ali et al. demonstrate via superresolution microscopy that immunolabeled IP_3_R, IRAG and PKG1 colocalize in a 40 nm nanoscale complex with the SR of isolated cerebral artery myocytes.^6^ Their close proximity in this signaling complex would allow PKG to rapidly phosphorylate IRAG and inhibit SR-mediated calcium release in response to elevated cGMP levels. Whether TRPM4 channels need to be expressed near this complex is not known.

An important remaining question is whether TRPM4 channels also mediate the vasodilatory effects of NO in other vascular beds. To date, studies on the role of TRPM4 channels in the regulation of myogenic tone have primarily been confined to the cerebral circulation^7^ and arterial myogenic responses have not been rigorously tested in TRPM4^−/−^ mice.^9^ It will be interesting to see if vasodilation through the NO/cGMP/PKG signaling pathway is altered in those mice, although global knock out animals may undergo genetic and physiologic compensation.^10^ The use of genetic strategies to manipulate TRPM4 and IRAG expression, preferably in a smooth muscle-specific manner, will presumably confirm and extend the findings of the present study. Additional work will also be required to determine whether this important mechanism for regulating vascular tone is specific to the mouse or also operates in other species, including humans.

## Funding

Funded by National Institutes of Health grant HL-122578.
